# *CmBES1* is a regulator of boundary formation in chrysanthemum ray florets

**DOI:** 10.1038/s41438-020-00351-8

**Published:** 2020-08-01

**Authors:** Peilei Cheng, Yanan Liu, Yiman Yang, Hong Chen, Hua Cheng, Qian Hu, Zixin Zhang, Jiaojiao Gao, Jiaxin Zhang, Lian Ding, Weimin Fang, Sumei Chen, Fadi Chen, Jiafu Jiang

**Affiliations:** State Key Laboratory of Crop Genetics and Germplasm Enhancement, Key Laboratory of Landscaping, Ministry of Agriculture and Rural Affairs, College of Horticulture, Nanjing Agricultural University, 210095 Nanjing, China

**Keywords:** Plant regeneration, Plant breeding

## Abstract

Chrysanthemum (*Chrysanthemum morifolium*) is an ideal model species for studying petal morphogenesis because of the diversity in the flower form across varieties; however, the molecular mechanisms underlying petal development are poorly understood. Here, we show that the brassinosteroid transcription factor *BRI1-EMS-SUPPRESSOR 1* (*CmBES1*) in chrysanthemum (*C. morifolium* cv. Jinba) is important for organ boundary formation because it represses organ boundary identity genes. Chrysanthemum plants overexpressing *CmBES1* displayed increased fusion of the outermost ray florets due to the loss of differentiation of the two dorsal petals, which developed simultaneously with the ventral petals. RNA-seq analysis of the overexpression lines revealed potential genes and pathways involved in petal development, such as *CUP-SHAPED COTYLEDON* (*CUC2*), *CYCLOIDEA 4* (*CYC4*), genes encoding MADS-box transcription factors and homeodomain-leucine zippers (*HD-Zips*) and auxin pathway-related genes. This study characterizes the role of *CmBES1* in ray floret development by its modulation of flower development and boundary identity genes in chrysanthemum.

## Introduction

Organogenesis is the process of tissue production by multipotent progenitor cells and is common to all multicellular organisms^1^. Lateral organs such as leaves and flowers are formed by lateral organ primordia, where cells are recruited from the periphery of the meristem^2^. In the central meristem, cell division maintains the stem cell population, while the growth of the surrounding cellular organ primordia is restricted and enters a quiescent state to form an organ boundary that separates itself from the central meristem and adjacent organs^3,4^. The boundary is important for organ shape because it allows for different growth patterns, and defects in the formation of organ boundaries can lead to organ-fusion phenotypes^5^.

Organ boundaries are regulated by complex networks comprising transcription factors and miRNAs and the spatial distribution of growth-promoting hormones such as auxin and brassinosteroids (BRs)^1,6^. The key genes involved in boundary regulation include *CUP-SHAPED COTYLEDON 1/2/3* (*CUC*), *LATERAL ORGAN FUSION* (*LOF*), *GROWTH REGULATING FACTOR* (*GRF*), and *LATERAL ORGAN BOUNDARY* (*LOB*), and the corresponding mutants of these genes have organ fusion phenotypes that contribute to our understanding of the importance of their roles in boundary regulation^7–10^. *CUC* genes encode *NAC* family transcription factors (*NAM*, *ATAF1/2*, and *CUC2*), which are regulated by miR164, and can control the expression of other boundary genes^7^, such as *KNAT6* (*KNOTTED*-like gene from *Arabidopsis thaliana*), which contributes to shoot apical meristem (SAM) maintenance and boundary establishment^11^. Organ boundaries are characterized by low cell expansion rates, and overexpression of *Arabidopsis thaliana homeobox 12* (*ATHB12*) leads to increased leaf cell expansion rates, indicating that *ATHB12* is a positive regulator of cell expansion^12^. Auxin concentration is higher in the meristem and developing primordia compared to that in the organ boundaries, where it limits the growth rate of boundary cells^13^. Spatial regulation of the brassinosteroid (BR) pathway is necessary for normal development of organ boundaries^6^. The *BRASSINAZOLE RESISTANT 1* (*BZR1*) and *BRI1-EMS SUPPRESSOR 1* (*BES1*) genes are components of the core regulators of BR signaling^14^. BR inhibits the expression of *CUC* at the organ boundary through transcriptional inhibition of the core transcription factor *BZR1*. A low level of *BZR1* in boundary cells ensures the appropriate expression of *CUC* and the normal formation of organ boundary shapes^5^. The development of lateral organs, such as flowers, is regulated by various transcription factors^15–18^. Specifically, the MADS-box family of transcription factors plays a major role in the control of flower architecture and induction^15^. Ectopic expression of *SUPPRESSOR OF OVEREXPRESSION OF CO1* (*GhSOC1*), which is an *AtSOC1*-*like1* MADS-box gene, in *Gerbera hybrida* led to a partial loss of floral organ identity^16^. *lfy* mutants, which have a mutation in the floral meristem identity gene *LEAFY* (*LFY*), show partial flower-to-shoot conversion^17^, and *AINTEGUMENTA-LIKE 6* (*AIL6*) is critical for cell differentiation in flowers^18^.

Chrysanthemum flowers are typical representatives of capitula, with many small individual flowers clustered at the top of the inflorescence axis. The shape resembles a large single flower but is actually composed of many flowers^19^. The capitulum contains two types of florets: outer ray florets and inner disc florets^20^. The ray florets are bilaterally symmetrical, with two dorsal petals that are degenerated and three fused petals that form an elongated ventral ligule, while the disc florets are radially symmetrical with five equal petals^21^. The shape of the ray floret is important for the chrysanthemum flower type and can be classified as flat, spoon, or tubular according to the degree of corolla tube merging (CTMD), which ranges from 0 to 0.2, 0.2 to 0.6, and 0.6 to 1.0 ^22^.

Many species in the Asteraceae family are ideal for the study of flower development. Mutants for inflorescence morphology, of which the *tubular-rayed* (*tub*) mutant of sunflower is a typical example^23^, have helped us to understand the floral traits of the capitulum. The *tub* mutant is caused by the loss of function of the *HaCYC2c* (*CYCLOIDEA-*like) gene, which leads to the generation of tubular ray florets^23^. Overexpression of *SvRAY2* (*CYC*-like genes) in *Senecio vulgaris* produced tubular ray florets^24^. In *G. hybrida*, *GhCYC2*, *GhCYC3*, and *GhCYC4* have redundant functions in regulating ray flower identity and promoting petal development in ray flowers^25^. In addition, *CmCYC2c* controls ray floret identity in chrysanthemum^20^.

Although the function of *BES1* in Arabidopsis has been well characterized, it is poorly understood in chrysanthemum. Here, we present evidence for the importance of *CmBES1* in the petal development of chrysanthemum ray florets. We show that when *CmBES1* is overexpressed in transgenic chrysanthemum plants, the two dorsal petals do not degenerate but instead develop simultaneously with the ventral petals and increase the degree of fusion of the outermost ray florets. RNA-seq analysis revealed that several genes associated with flower development were differentially expressed, including *CUC2*, *CYC4*, genes encoding MADS-box transcription factors and HD-ZIP proteins, and auxin pathway-related genes. Overall, the present study links *CmBES1* to ray floret development through the regulation of flower development and organ boundary identity gene expression in chrysanthemum.

## Results

### *CmBES1* sequence characteristics

**Fig. 1:**
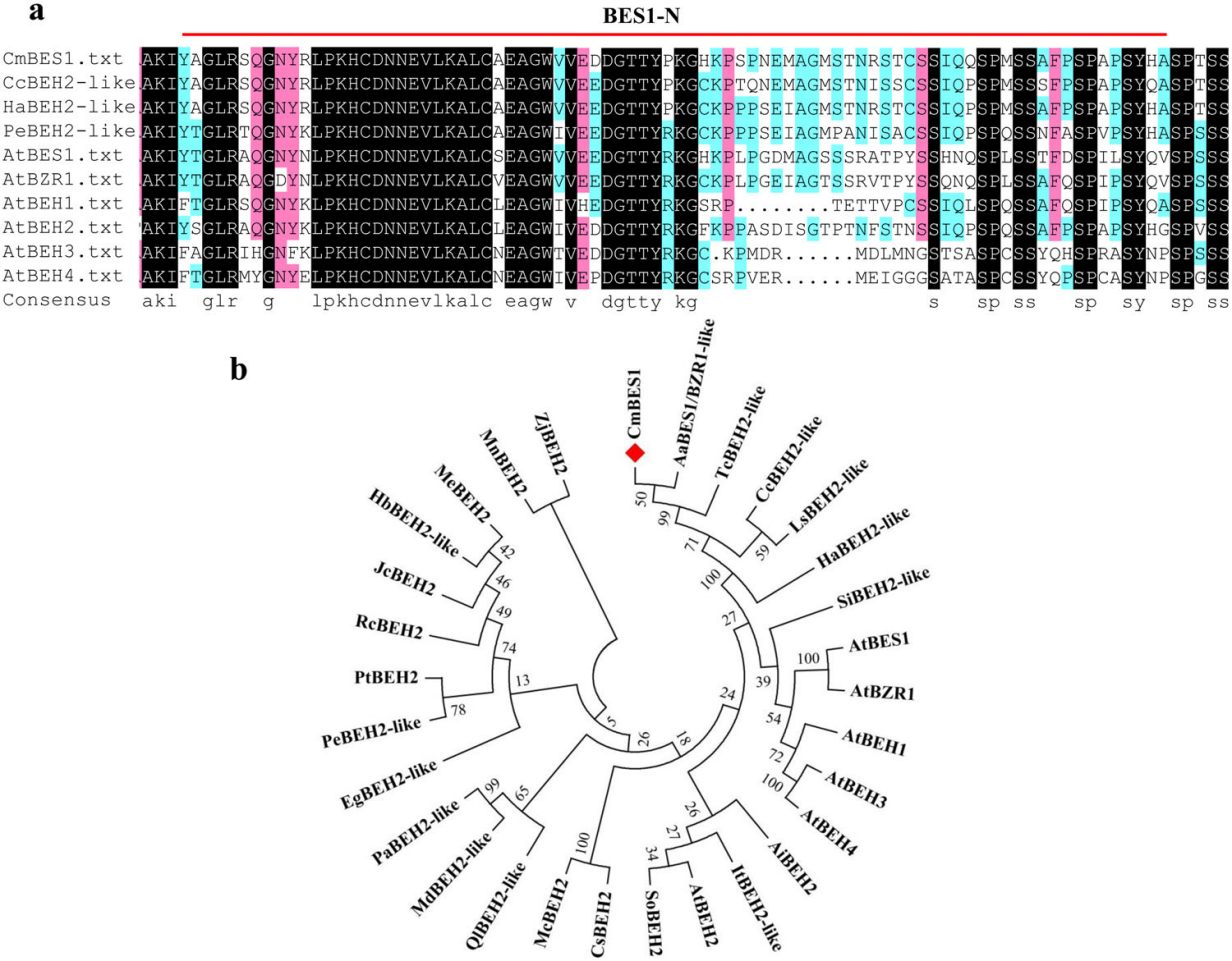
Deduced amino acid sequence comparison and phylogenetic analysis of *CmBES1*. **a** Amino acid sequence alignment of CmBES1 with BES1 sequences from various plant species. The single red lines indicate the conserved BES1-N region in BES1-like proteins. The black color indicates 100% identity; red, 75% identity; and blue, 50% identity. The sequences compared to CmBES1 were *Arabidopsis thaliana AtBES1* (AT1G19350), *AtBZR1* (AT1G75080), *AtBEH1* (AT3G50750.1), *AtBEH2* (AT4G36780), *AtBEH3* (AT4G18890), and *AtBEH4* (AT1G78700); *Helianthus annuus* BES1/BZR1 homolog protein 2-like (*HaBEH2-like*, LOC110908961); *Cynara cardunculus* BES1/BZR1 homolog protein 2-like (*CcBEH2-like*, LOC112512677); and *Populus euphratica* BES1/BZR1 homolog protein 2-like (*PeBEH2-like*, LOC105107647). **b** Phylogenetic tree comprising the following *CmBES1* and BES1 family proteins: *Arabidopsis thaliana AtBES1* (AT1G19350), *AtBZR1* (AT1G75080), *AtBEH1* (AT3G50750.1), *AtBEH2* (AT4G36780), *AtBEH3* (AT4G18890), and *AtBEH4* (AT1G78700); *Arachis ipaensis AiBEH2* (XP_016201823.1); *Artemisia annua AaBES1/BZR1-like* (PWA37330.1); *Cucumis sativus CsBEH2* (XP_004143497.1); *Cynara cardunculus CcBEH2-like* (LOC112512677); *Eucalyptus grandis EgBEH2-like* (NP_001306904.1); *Helianthus annuus HaBEH2-like* (LOC110908961); *Hevea brasiliensis HbBEH2-like* (XP_021646974.1); *Ipomoea triloba ItBEH2-like* (XP_031095547.1); *Jatropha curcas JcBEH2* (XP_012079081.1); *Lactuca sativa LsBEH2-like* (XP_023759843.1); *Malus domestica MdBEH2-like* (XP_008338783.2); *Manihot esculenta MeBEH2* (XP_021632984.1); *Momordica charantia McBEH2* (XP_022132946.1); *Morus notabilis MnBEH2* (XP_024020611.1); *Populus euphratica PeBEH2-like* (LOC105107647); *Populus trichocarpa PtBEH2* (XP_002310201.1); *Prunus avium PaBEH2-like* (XP_021809451.1); *Quercus lobata QlBEH2-like* (XP_030949816.1); *Ricinus communis RcBEH2* (XP_002525100.1); *Sesamum indicum SiBEH2-like* (XP_011097209.1); *Spinacia oleracea SoBEH2* (XP_021835401.1); *Tanacetum cinerariifolium TcBEH2-like* (GEY43666.1); and *Ziziphus jujuba ZjBEH2* (XP_015890203.1). The phylogenetic tree was constructed using the neighbor-joining method and bootstrap test with 1000 replicates. The divergence of each branch is indicated by the bootstrap values

To better understand the effects of *BES1* family genes on chrysanthemum, a homolog of the *BES1* gene was identified. The genomic sequence of *CmBES1* was cloned from leaves of *C. morifolium* cv. Jinba; the gene had a predicted open reading frame (ORF) of 921 bp and encoded a 306-amino acid protein. A BLAST nucleotide sequence search of *CmBES1* in The Arabidopsis Information Resource (TAIR; http://www.arabidopsis.org/) revealed that the *BES1* family of genes in Arabidopsis most closely related to *CmBES1* was the *AtBES1* family. The deduced polypeptide of CmBES1 was similar in various plant species and had a highly conserved BES1-N domain in the N terminus (Fig. 1a, Supplementary Fig. S1). Phylogenetic analysis (Fig. 1b) showed that CmBES1 and AaBES1/BZR1-like were closely related, with an amino acid sequence similarity of 71.12%. The levels of peptide identity between CmBES1 and other proteins were 70.48% (TcBEH2-like), 66.83% (HaBEH2-like), 65.16% (CcBEH2-like), and 45.11% (AtBES1).

### Subcellular localization, expression patterns, and transcriptional activity analysis of CmBES1

**Fig. 2:**
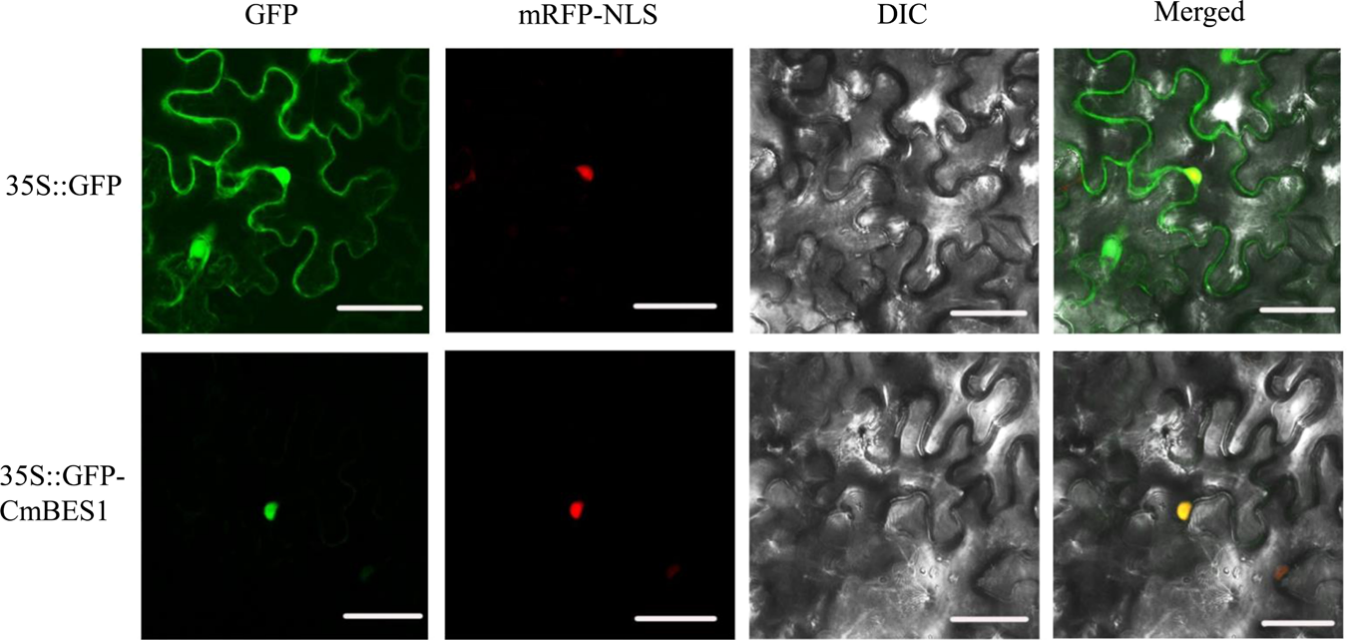
Subcellular localization of CmBES1 in *N. benthamiana* epidermal cells. The images correspond to the GFP (left), mRFP-NLS, DIC (middle), and merged (right). 35S::*D53*-RFP was used as a nuclear marker (mRFP-NLS). Bars: 50 μm

To examine the subcellular localization of CmBES1, a 35S::GFP-*CmBES1* construct was developed and introduced into *Nicotiana benthamiana* epidermal cells, along with a 35S::GFP construct as a control. The 35S::GFP construct alone was detected in both the cytoplasm and the nucleus of the tobacco cells (Fig. 2), while the CmBES1-GFP fusion protein was detected only in the nucleus. These data suggest that CmBES1 is localized in the nucleus.

**Fig. 3:**
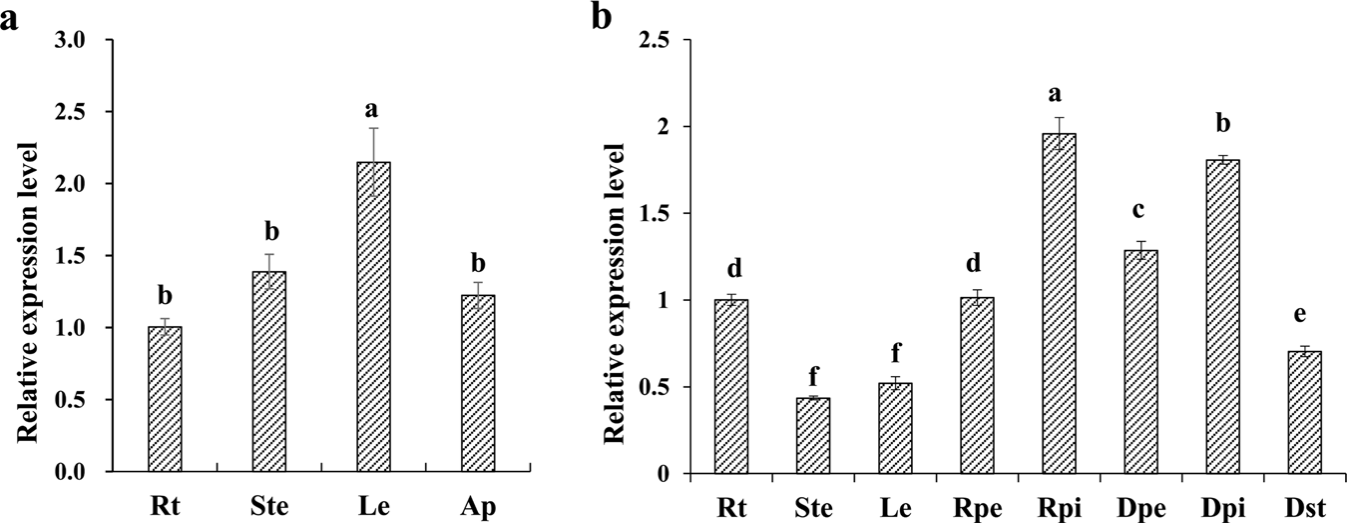
Expression pattern analysis of *CmBES1*. **a** Expression patterns of *CmBES1* during vegetative growth in the roots (Rt), stems (Ste), leaves (Le) and shoot apex (Ap). **b***CmBES1* expression patterns during the reproductive stage in the roots (Rt), stems (Ste), leaves (Le), ray floret petals (Rpe), ray floret pistil (Rpi), disc floret petals (Dpe), disc floret pistil (Dpi), and disc floret stamens (Dst). EF-1α expression in chrysanthemum was used as an internal control. The error bars indicate SEs (*n* = 3). The differences were analyzed by Duncan’s multiple range test. The different lowercase letters indicate significant differences (*P* < 0.05)

We further examined changes in *CmBES1* gene expression by quantitative real-time polymerase chain reaction (qRT-PCR) during vegetative and reproductive periods of Jinba chrysanthemum plant organs to predict their possible functions (which have not been predicted) (Fig. 3). *CmBES1* transcripts were detected in all organs at all developmental stages. During the vegetative period, the transcript level of *CmBES1* was abundant in the leaves and showed similar expression in the roots, stems, and shoot apex (Fig. 3a). During reproductive growth, the transcript level of *CmBES1* was high in the ray floret pistils and disc floret pistils; moderate in the ray floret petals, disc floret petals, and roots; and low in the stems, leaves, and disc floret stamens (Fig. 3b).

**Fig. 4:**
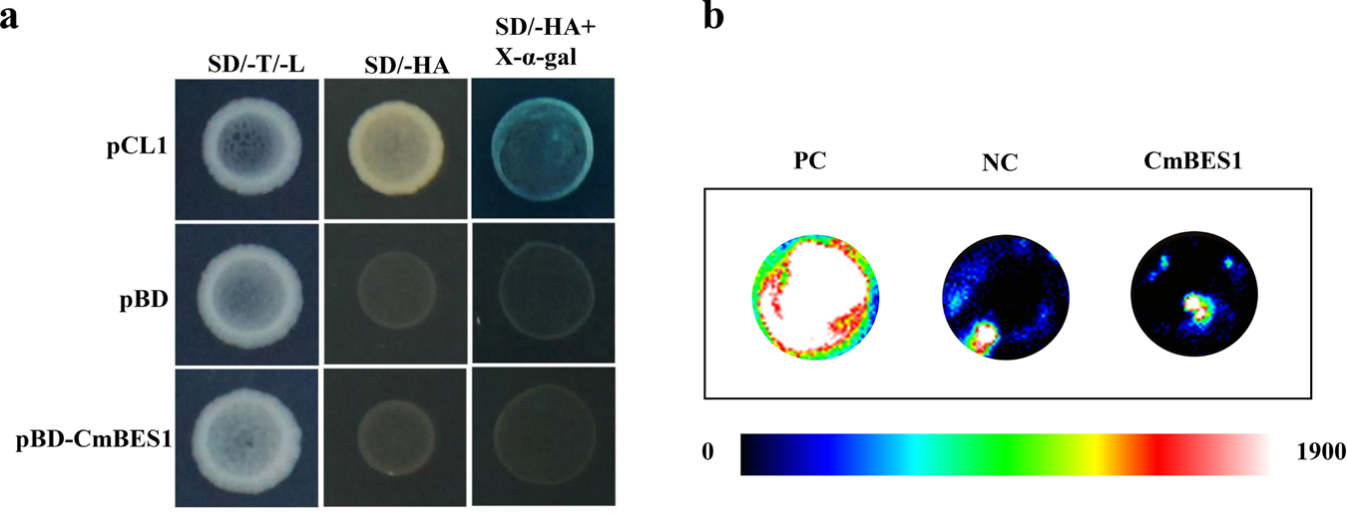
Analysis of *CmBES1* transactivation. **a** Transcriptional activity of *CmBES1* measured in a yeast assay system. *Y2H* cells expressing pCL1 grew on SD/-His-Ade media and served as positive controls. *Y2H* cells expressing pGBKT7 could not grow on this media and served as negative controls. SD/-T/-L: SD/-Trp media (pGBKT7 and genes) or SD/-Leu media (pCL1), SD/-HA: SD/-His/-Ade media, SD/-HA + X-α-gal: SD/-His/-Ade media+X-α-gal. **b** Relative luciferase activities in Arabidopsis mesophyll protoplasts after transfection with 35 S::GAL4DB-*CmBES1*; the 35 S::GAL4DB-*AtARF5* construct served as a positive control, and 35 S::GAL4DB served as a negative control

To test the transactivation activity of the CmBES1 protein, a transactivation assay was performed in yeast. Yeast expressing the pCL1 plasmid grew well on SD/-His-Ade media and turned blue on SD/-His-Ade media supplemented with X-a-gal (Fig. 4a); however, the negative control pGBKT7 and pGBKT7-*CmBES1* constructs did not turn blue on this selective medium. To further confirm these results, we transfected CmBES1 as an effector plasmid into Arabidopsis protoplasts, and the results showed that the relative luciferase (LUC) activity of 35S::GAL4DB-*AtARF5* was significantly higher than that of 35S::GAL4DB-*CmBES1* (Fig. 4b, Supplementary Fig. S2, *P* < 0.01). The relative LUC activity of 35S::GAL4DB-*CmBES1* was lower than that of 35S::GAL4DB but was not significantly different (Fig. 4b, Supplementary Fig. S2), indicating that *CmBES1* acts as a repressor of transcription.

### *CmBES1* is involved in chrysanthemum petal development

**Fig. 5:**
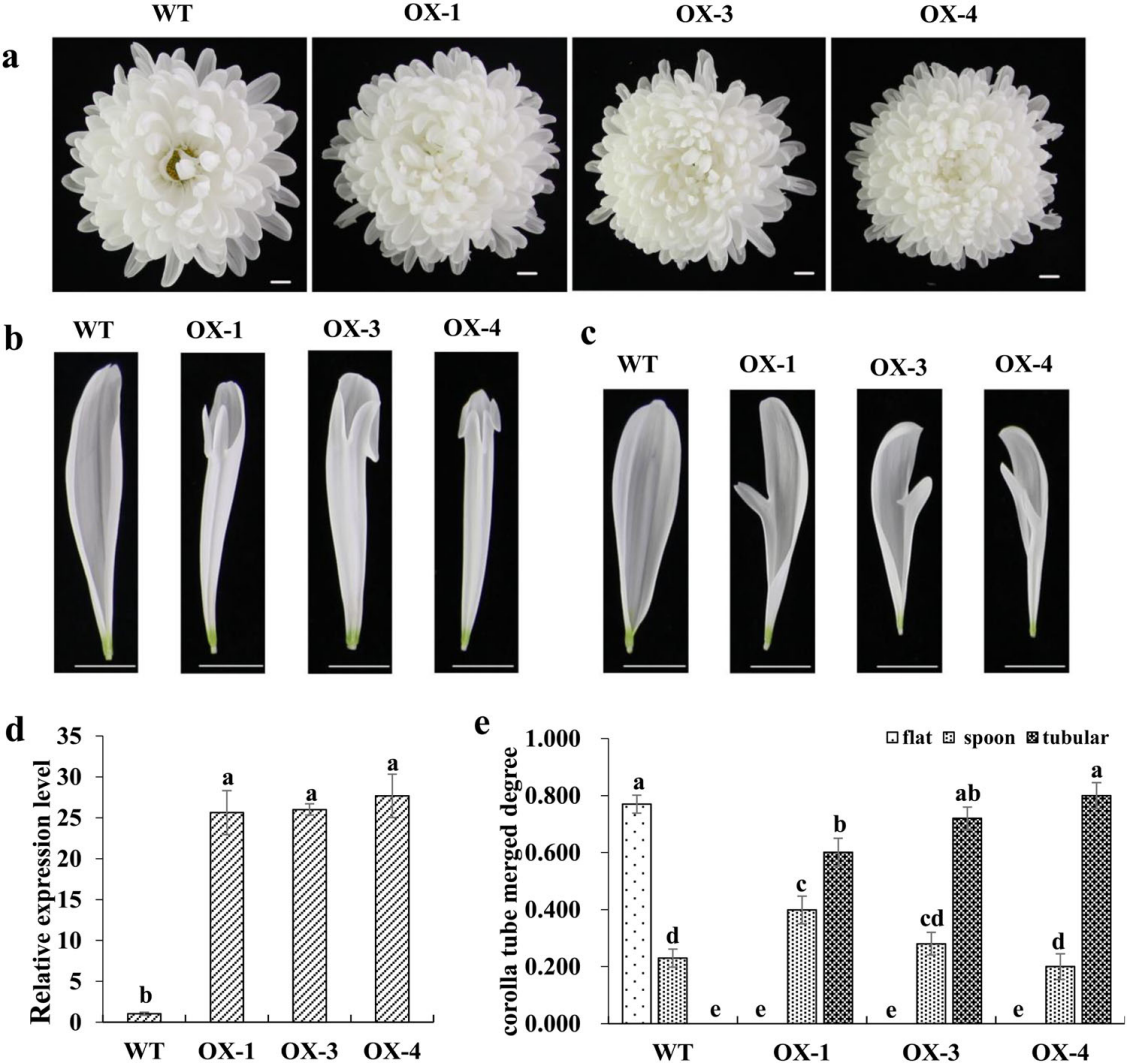
Ectopic expression of *CmBES1* affected the corolla tube merged degree. **a** Top view of wild type (WT) and transgenic overexpression (OX) lines 1, 3, and 4. The positive transgenic lines had a relatively high degree of corolla tube merging (corolla tube length/ray floret length). Bar: 1 cm. **b** Top view of the outermost ray florets of WT and OX lines. Bar: 1 cm. **c** Top view of the middle ray florets of WT and OX transgenic plants. Bar: 1 cm. **d** Expression level of *CmBES1* in WT and OX transgenic plants. The error bars indicate SEs (*n* = 3). The differences were analyzed by Duncan’s multiple range test. The different lowercase letters indicate significant differences (*P* < 0.05). **e** Corolla tube merged degree between the outermost ray florets. The error bars indicate SEs (*n* > 100). The differences were analyzed by Duncan’s multiple range test. The different lowercase letters indicate significant differences (*P* < 0.01)

To further investigate the function of *CmBES1* in chrysanthemum growth, a population of seven *CmBES1*-overexpressing lines was validated by confirming the increase in *CmBES1* expression in transgenic lines compared with wild-type (WT) plants by qRT-PCR analysis (Supplementary Fig. S3), and three representative *CmBES1*-overexpressing lines (OX-1, OX-3, and OX-4) were selected for functional analysis (Supplementary Fig. S3, Fig. 5d). We evaluated the inflorescences of OX-*CmBES1* transgenic plants and found that the ray floret shape was different from that of WT plants (Fig. 5a–c). The CTMD of the outermost ray florets increased, while the degree of the middle ray floret fusion did not change; however, the shape of the petals was altered at the tips of ray florets, and the petal tips exhibited petalized protrusions (Fig. 5a–c). To further study the changes in the degree of merging of the outermost ray florets, 15 of the outermost ray florets from each inflorescence of 15 individual plants were randomly selected from each transgenic line to measure the degree of ray floret fusion. The chrysanthemum ray floret shape can be flat, spoon shaped, or tubular, with the CTMD ranging from 0 to 0.2 (flat), 0.2 to 0.6 (spoon shaped), and 0.6 to 1.0 (tubular)^22^. Among the WT chrysanthemum plants, 77.0% of the total number of the outermost ray florets were flat, and 23.0% were spoon shaped; there were no tubular outermost ray florets (Fig. 5e). In transgenic overexpression lines, the outermost ray florets were mainly tubular. The proportion of spoon ray florets in the transgenic lines ranged from 20.0 to 39.9%, and the proportion of tubular ray florets ranged from 60.1 to 80.0%.

**Fig. 6:**
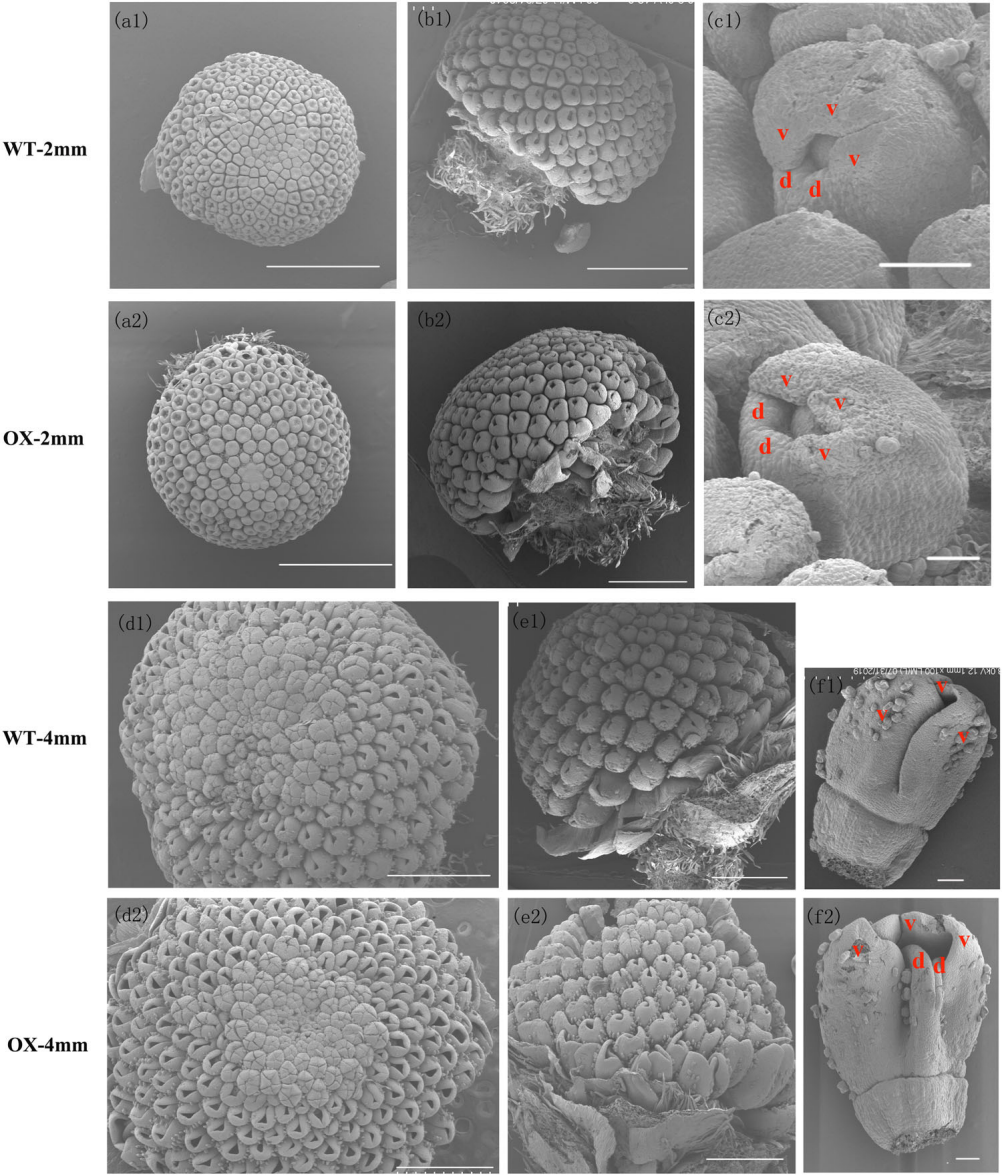
Morphological analysis of the inflorescence of the wild type and *CmBES1*-overexpressing transgenic lines at the 2 and 4 mm stages. **a1**, **a2** Top view of the inflorescence at the 2 mm stages. Bar: 1 mm. **b1**, **b2** Side view of the inflorescence at the 2 mm stage. Bar: 1 mm. **c1**, **c2** SEM images of ray florets at the 2 mm stage. Bar: 100 μm. d and v in red letters represent dorsal and ventral petals, respectively. **d1**, **d2** Top view of the inflorescence at the 4 mm stage. Bar: 1 mm. **e1**, **e2** Side view of the inflorescence at the 4 mm stage. Bar: 1 mm. **f1**, **f2** SEM images of ray florets at the 4 mm stage. Bar: 100 μm. d and v in red letters represent dorsal and ventral petals, respectively. Ectopic expression of *CmBES1* increased the corolla tube merged degree

To detect the onset of this different phenotype, images of the OX-4 transgenic line were taken with a scanning electron microscope (SU8010 device, Hitachi, Japan). A whole inflorescence (Fig. 6a1, a2, b1, b2) or only the outermost ray florets were sampled when inflorescences were ~2 and ~4 mm long. When inflorescences whose diameter was ~2 mm were sampled, the OX-4 transgenic outermost ray florets were not significantly different from those of the WT (Fig. 6c1, c2). Petals were sampled when their diameter was ~4 mm (Fig. 6f1, f2). In the WT line, the peripherally located ray florets were bilaterally symmetrical to two rudimentary dorsal petals and an elongated ventral ligule was formed by three fused petals. In the transgenic line, the two dorsal petals did not degenerate and developed simultaneously with the ventral ligule, which increased the degree of fusion of the outermost ray florets. We also imaged the fresh samples at the ~4, ~6, and ~8 mm stages as well as the relevant petals using light microscopy (Supplementary Fig. S4) to evaluate their development, and characterize the fusion phenotype of the transgenic chrysanthemum plants. The degree of fusion of the outermost ray florets was increased in transgenic lines compared with the WT. These results suggested that overexpression of *CmBES1* affects the degree to which the ray florets fused to form the corolla tube and alters the pattern of the outermost ray florets.

### Global expression of downstream *CmBES1*-associated transcripts in chrysanthemum outermost ray florets

To better understand the mechanisms by which *CmBES1* mediates the regulation of floral development, a large-scale screen of genes differentially expressed in WT and OX-4 transgenic lines was performed using RNA sequencing (RNA-seq). The RNA from the outermost ray florets of WT and OX-4 plants was sampled from inflorescences with a diameter of ~4 mm. A summary of the sequence reads is given in Supplementary Table S1, with the contents of each library ranging from 62.74 to 65.52 million clean reads. After assembly, the outcome involved a set of 98,155 unigene sequences with a mean length of 987 nucleotides (nt); the N50 (genome splicing quality) was 1407 nt. The total number of unigenes obtained from the six libraries (WT-1, WT-2, WT-3, OX4-1, OX4-2, and OX4-3) was 65,984, 59,431, 62,636, 67,405, 64,908, and 65,476, respectively. Differentially expressed genes (DEGs) were identified from pairwise comparisons between WT and OX-4, with a false discovery rate (FDR) less than 0.05. A total of 9722 DEGs were obtained from the transcriptomic data, of which 4986 were upregulated and 4736 were downregulated (Supplementary Fig. S5a). In addition, NR species distribution, KEGG pathway classification, and GO classification analyses were performed (Supplementary Fig. S5).

Based on the *CmBES1* regulation of the CTMD of the ray florets, we focused our analysis on the DEGs related to organ boundary growth and flower development (Supplementary Table S2), which revealed genes related to the auxin pathway, such as the homologs of *AUXIN RESPONSE FACTOR* (*ARF*) and *INDOLEACETIC ACID-INDUCED PROTEIN* (*IAA*). The expression levels of some boundary genes varied between the WT and overexpression lines; the central boundary gene is a homolog of the *Helianthus annuus* protein *CUP-SHAPED COTYLEDON 2-like* (CL12058.Contig3_All), which was downregulated in OX-4 compared to WT. Additionally, other boundary-related genes, such as *CmKNAT2* (CL3389.Contig2_All) and the WUSCHEL-related homeobox homologs *WOX3* (CL9508.Contig2_All) and *WOX4* (Unigene1685_All), were also downregulated in OX-4. *CmCYC2* controls ray floret identity in chrysanthemum^20^, and we found the *CmCYC4* homolog (CL4616.Contig3_All) also had relatively low expression, suggesting that it also plays a role in regulating the degree of chrysanthemum ray floret fusion. The MADS-box transcription factor plays an important role in flower development because it regulates the characteristics of floral organs and influences their subsequent development^26^. Homologs of the Agamous-like MADS-box protein and MADS-box transcription factor were differentially expressed, with *CmBES1* being overexpressed. Homeodomain-leucine zipper (*HD-Zip*) genes that contain homeodomains with highly conserved leucine zipper motifs are unique to plants^27^. *Arabidopsis thaliana homeobox 12* (*ATHB12*) is an *HD-Zip I* gene that is mainly expressed in leaves and stems and represses the expression of *gibberellin 20 oxidase 1* (*GA20ox1*), thereby inhibiting stem elongation during the early development of inflorescence stems^12^. Five chrysanthemum *homeobox* (*HB*) genes were found in our transcriptomic data, as well as other genes related to flower development that were also evaluated.

**Fig. 7:**
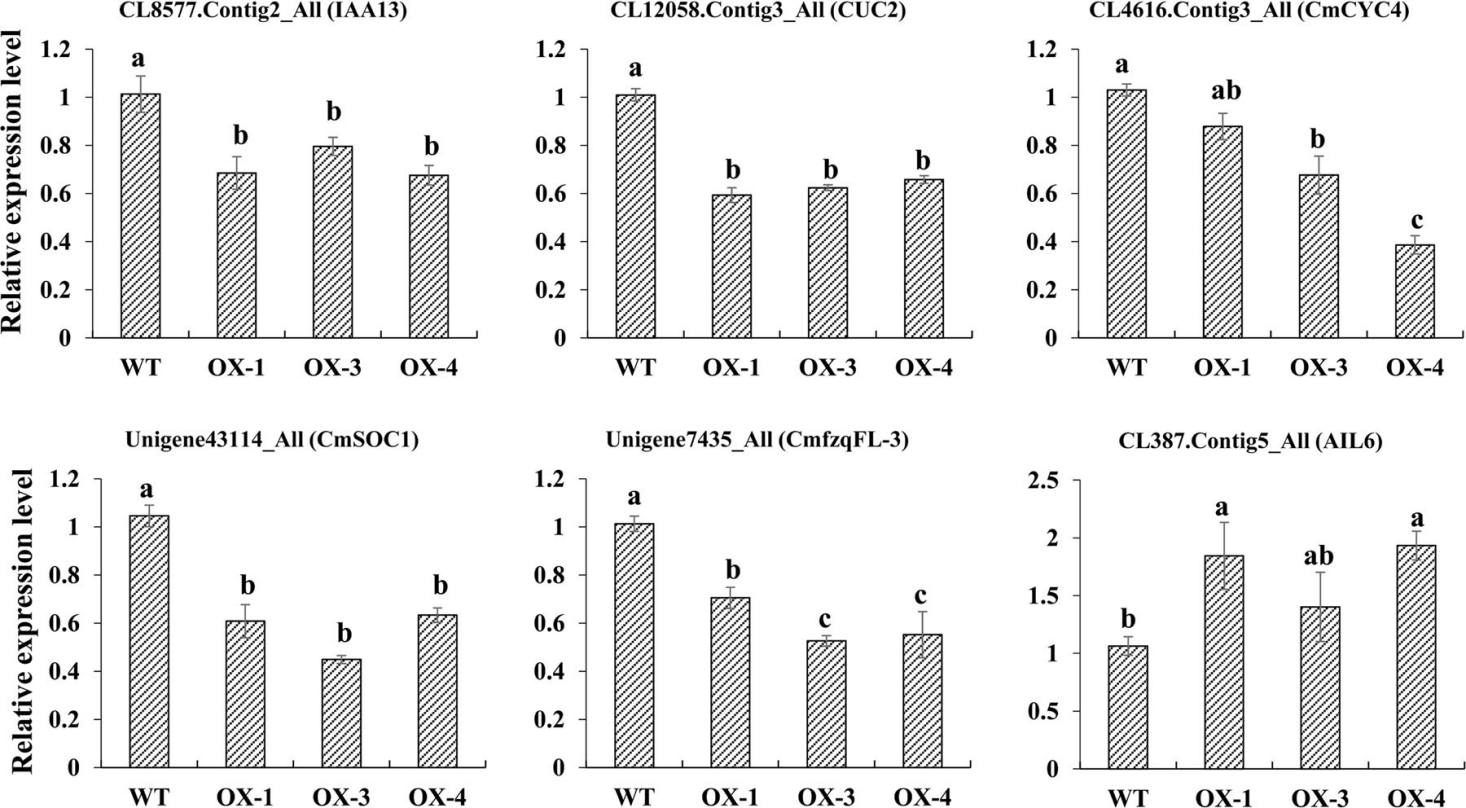
Validation of the RNA-seq classification of differential transcription via qRT-PCR. The error bars indicate SEs (*n* = 3). The differences were analyzed by Duncan’s multiple range test. The different lowercase letters indicate significant differences (*P* < 0.05)

The above results led us to examine the expression of genes that can serve as markers for ray floret petal development. The expression of several identified genes, such as *CUC2*, *CYC4*, *SOC1-like*, *CmfzqFL-3*, *AIL6*, and *IAA13*, was confirmed by qRT-PCR (Fig. 7). RNA was prepared from the outermost ray florets of WT, OX-1, OX-3, and OX-4 samples collected from florets whose diameter was ~4 mm. Although the expression fold changes indicated by RNA-seq and qRT-PCR differed in detail, the overall sequencing data were reliable (Fig. 7).

## Discussion

Boundary formation is critical to the function of mature organs because it allows for correct patterning and separation of different activities. These boundaries are regulated by complex networks that include transcription factors and the spatial distribution of growth-promoting hormones such as auxin and BRs^1,6^. In Arabidopsis, the core gene involved in BR signaling and the node of multiple signal cascades is *BZR1*, which is closely related to organ fusion. Indeed, the *bzr1-1D* mutant has general defects in various organ boundaries, such as stems that bend toward the axillary branches and cauline leaves, bending of siliques at the silique-pedicel junction, and fused stamens; however, its petals develop normally^5^. Little is known about the genetic and molecular mechanisms underlying the key transcription factor regulating BR signaling during organ development in chrysanthemum. We showed that the chrysanthemum *BES1* gene is involved in regulating the fusion of the ray floret corolla but does not affect the development of other organs, which is significantly different from the fusion regulated by *BZR1* in *Arabidopsis thaliana*^5^. Overexpression of *CmBES1* in transgenic chrysanthemum plants promoted an increased degree of fusion of the outermost ray florets because the two dorsal petals developed simultaneously with the ventral ligule rather than differentiating. In the WT cut flower chrysanthemum Jinba, more than 70% of the outermost ray florets are flat, and only part of the higher degree of fusion presents a spoon-like phenotype. The transgenic overexpression line does not produce flat outermost ray florets; instead, they are mainly tubular. The pattern of *CmBES1* accumulation in the ray floret pistil is consistent with its inhibitory role in boundary formation and its activation role during cell growth promotion.

To gain a better understanding of how *CmBES1* affects organ development, we conducted RNA-seq-based transcript profiling to identify potential genes and pathways involved in organ fusion. In the present study, RNA-seq-based transcript profiling showed that the expression levels of a number of gene homologs to known components of flower development and boundary regulation were differentially expressed in transgenic chrysanthemum overexpressing *CmBES1* compared with those of WT plants. In Arabidopsis, mutants with increased BR signaling showed severe organ fusions across a wide range of boundary tissues. These phenotypes are caused by *BZR1* suppression of the organ boundary identity genes *CUC* and *LOF*. *BZR1* is mainly expressed in the SAM but is expressed at low levels at the boundary, which ensures the proper expression of boundary identity genes during normal boundary formation^5^. *BES1* in chrysanthemum is a typical transcription factor with the highest homology with *AtBES1*, compared with other *BES1* family genes (*AtBEH1-AtBEH4*, *AtBZR1*) in Arabidopsis. Its subcellular localization is in the nucleus, with no transcriptional activation activity, and mainly in the ray floret pistil during the reproductive growth period. The activity pattern of *CmBES1* is similar to that of Arabidopsis *BZR1*; *CmBES1* inhibits *CUC2* transcription, causing the downregulation of *CUC2* at the boundary of ray florets and the appearance of the petal fusion phenotype and architectural alteration. As the dorsal petals of the ray florets elongated dramatically, the expression of several genes related to flower development also changed significantly in the transgenic chrysanthemum plants. MADS-box genes play a major role in the control of flower architecture^15^; for example, *GhSOC1* in *G. hybrida* controls floral organ identity^16^, and the *APETALA 1* (*AP1*) gene in Arabidopsis, which has an A-class homeotic function, is required for sepal and petal development^28^. Homologs of MADS-box genes (*SOC1-like*, *AP1-like*) showed significant changes in expression in transgenic chrysanthemum. We also identified several HD-ZIP genes; given the role of *ATHB12* in cell expansion^12^, it is tempting to speculate that the identified chrysanthemum HD-ZIP protein may function in the regulation of petal development (Supplementary Table S2). Of primary importance for flower primordium initiation is the ARF MONOPTEROS protein (MP/ARF5); MP induces the expression of *LFY*, which specifies floral fate, and of two ANT/AIL6 transcription factors, which are key regulators of floral growth^29^. RNA-seq-based transcript profiling suggests that the roles of the *LFY-like* (*CmfzqFL-3*), *AIL6*, and *ARF-like* genes are significantly altered in transgenic chrysanthemum and could be related to the fusion of the ray floret corolla. These findings suggest that *CmBES1* expression influences ray floret development by modulating flower development and the expression of genes related to boundary regulation.

In addition to the changes in ray floret petal development, we also found that the number of ray florets in transgenic *CmBES1*-OX lines is different significantly from those of the WT (Supplementary Fig. S6). There was no significant difference in the total number of florets in the capitulum between WT and OX lines; however, in the transgenic lines, the number of disc florets was significantly decreased compared to those in the WT, but the number of ray florets significantly increased, indicating that the *BES1* expression level affects ray floret identity. Previous studies have shown that variation in the capitulum type is dependent on the replacement of ray florets by disc florets or vice versa^30^. The phenotype of the fusion of the ray floret corolla in transgenic chrysanthemum plants overexpressing *CmBES1* is similar to the phenotype of the *tubular-rayed* (*tub*) mutant of sunflower, while the phenotype of disc florets that turn into ray florets is similar to that of the *double-flowered* (*dbl*) sunflower mutants^23^. Research has shown that misexpression of the *HaCYC2c* gene causes a *dbl* phenotype, whereas loss of gene function causes a *tub* phenotype^23^. In fact, the *CYC* gene has been widely studied in the Asteraceae family, and overexpression of *GhCYC2* in *G. hybrida* causes disc florets to present a morphology similar to that of ray florets^25^. However, in *S. vulgaris*, overexpression of *SvRAY1* (a *CYC-like* gene) repressed ray floret development and overexpression of *SvRAY2* produced tubular ray florets^24^. Transgenic analysis showed that overexpression of *CmCYC2c* in chrysanthemum led to a significant increase in the number of ray florets per inflorescence compared to the number in WT^20^. Further molecular analysis suggests that *CmCYC2-like* transcription factors may interact with each other or bind to the promoter to regulate floral symmetry development in *Chrysanthemum morifolium*^31^. Differences in transgenic phenotypes in the Asteraceae family suggest that the *CYC* gene may play a role in promoting or inhibiting the development of ray florets. The results indicate that *CYC* plays a critical role in controlling ray flower identity and floral organ development in the Asteraceae family. RNA-seq analysis showed that the *CYC* homolog *CYC4* was downregulated in the transgenic *CmBES1*-OX lines, suggesting that it might be a new candidate gene that regulates ray flower identity and petal development. The relationship between *CmBES1* and *CYC4* and their regulation of inflorescence development will need further investigation.

Some genes involved in the BR biosynthetic pathway are suppressed by the final product of the BR signaling pathway through a feedback loop, such as *CYP90B1* (*DWF4*), *Constitutive photomorphogenesis and dwarfism* (*CPD*), *CYP85A1* (*BR6ox1*), and *Rotundifolia 3* (*ROT3*), which are downregulated by *BZR1* when it directly binds to the promoter region of these genes^32^. Through further mining of the transcriptomic data, we found that the expression level of *CPD* (CL5486.Contig2_All) was upregulated, while the expression levels of BR6ox1 (CL13153.Contig1_All) homologs and *ROT3* (Unigene11129_All) were slightly upregulated but did not significantly differ (Supplementary Table S3, Supplementary Fig. S7). Based on the above data, we found that *CmBES1* feedback regulates the expression of BR synthesis genes in chrysanthemum, suggesting that this gene is similar to the homologous gene of Arabidopsis but that there are some differences in its regulation of downstream effectors that may be species specific.

Our findings provide insight into the mechanism of ray flower identity and petal development through *CmBES1*, which regulates flower development and boundary identity genes in chrysanthemum. This study provides important evidence for the molecular mechanism underlying inflorescence development and is potentially relevant for molecular breeding strategies.

## Materials and methods

### Plant materials and growth conditions

Samples of the *C. morifolium* cv. Jinba, which is a popular variety as a cut flower on the market, was obtained from the Chrysanthemum Germplasm Resource Conservation Centre (Nanjing Agricultural University, China). The plants were cultured in a greenhouse under a 16-h light/8-h dark photoperiod (80–100 μmol/m^2^/s irradiation) with 70% relative humidity and a day/night temperature of 26/15 °C. Petals were sampled from the outermost whorl of the inflorescence and used for both RNA extraction and morphological analysis.

### Cloning and phylogenetic analysis of *CmBES1*

Total RNA was extracted from leaves and used for cDNA synthesis as previously described^33^. A *CmBES1*-specific primer pair (BES1-F and -R, Supplementary Table S4) was designed with Primer 5.0 software (www.bbioo.com/Soft/2005/114.htm) according to the Unigene19979 sequence in the Jinba chrysanthemum transcriptome to amplify the ORF. The resulting PCR product was purified and cloned into pMD19-T (TaKaRa, Tokyo, Japan) for sequencing. Homologs of *BES1* in other species were obtained using BLAST searches (https://blast.ncbi.nlm.nih.gov/Blast.cgi), and the *BES1* transcription factor family members in Arabidopsis were obtained from TAIR (http://www.arabidopsis.org/). Phylogenetic trees were constructed using MEGA 5.0 software with the neighbor-joining method and bootstrap test using 1000 replicates^34^. The *BES1* family genes comprised the following: *Arabidopsis thaliana AtBES1* (AT1G19350), *AtBZR1* (AT1G75080), *AtBEH1* (AT3G50750.1), *AtBEH2* (AT4G36780), *AtBEH3* (AT4G18890), and *AtBEH4* (AT1G78700); *Arachis ipaensis AiBEH2* (XP_016201823.1); *Artemisia annua AaBES1/BZR1-like* (PWA37330.1); *Cucumis sativus CsBEH2* (XP_004143497.1); *Cynara cardunculus CcBEH2-like* (LOC112512677); *Eucalyptus grandis EgBEH2-like* (NP_001306904.1); *Helianthus annuus HaBEH2-like* (LOC110908961); *Hevea brasiliensis HbBEH2-like* (XP_021646974.1); *Ipomoea triloba ItBEH2-like* (XP_031095547.1); *Jatropha curcas JcBEH2* (XP_012079081.1); *Lactuca sativa LsBEH2-like* (XP_023759843.1); *Malus domestica MdBEH2-like* (XP_008338783.2); *Manihot esculenta MeBEH2* (XP_021632984.1); *Momordica charantia McBEH2* (XP_022132946.1); *Morus notabilis MnBEH2* (XP_024020611.1); *Populus euphratica PeBEH2-like* (LOC105107647); *Populus trichocarpa PtBEH2* (XP_002310201.1); *Prunus avium PaBEH2-like* (XP_021809451.1); *Quercus lobata QlBEH2-like* (XP_030949816.1); *Ricinus communis RcBEH2* (XP_002525100.1); *Sesamum indicum SiBEH2-like* (XP_011097209.1); *Spinacia oleracea SoBEH2* (XP_021835401.1); *Tanacetum cinerariifolium TcBEH2-like* (GEY43666.1); and *Ziziphus jujuba ZjBEH2* (XP_015890203.1). The conserved domain of the CmBES1 sequence was inferred by querying the Conserved Domains Database (www.ncbi.nlm.nih.gov/Structure/cdd/wrpsb.cgi), and DNAMAN software was used for multiple sequence alignment.

### Subcellular localization, expression patterns, and transcriptional activity analysis of CmBES1

The amplicon of CmBES1 and the pENTR 1A vector (Invitrogen, Carlsbad, CA, USA) were digested with *Sal*I and *Eco*RI endonucleases (the primers used are listed in Supplementary Table S4) and then ligated with T4 DNA ligase (TaKaRa, Tokyo, Japan) before sequencing. The plasmid pENTR1A-*CmBES1* was recombined with the binary vector pMDC43 (35S::GFP) using an LR reaction (Invitrogen, Carlsbad, CA, USA) to obtain a 35S::GFP-*CmBES1* green fluorescent protein fusion construct^35^. The 35S::GFP-*CmBES1* and free GFP (35S::GFP) plasmids were transiently coexpressed together with a nuclear marker (35S::*D53*-RFP construct)^36^ into *Nicotiana benthamiana* leaves. The leaf epidermal cells were monitored with a Zeiss LSM 780 confocal microscope (Zeiss, Jena, Germany) after 3 days.

Temporal and spatial expression characteristics of *CmBES1* during the vegetative and reproductive periods were analyzed. Samples from the vegetative period comprised roots (Rt), stems (Ste), leaves (Le), and the shoot apex (Ap), and were collected from rooted chrysanthemum seedlings at the 9–10 leaf stage grown under LDs (16 h light/8 h dark). Samples from the reproductive period comprised Rt, Ste, Le, ray floret petals (Rpe), ray floret pistil (Rpi), disc floret petals (Dpe), disc floret pistil (Dpi), and disc floret stamens (Dst) and were collected from WT chrysanthemum plants at the early opening stage. The *EF-1α* (GenBank: AB548817.1) gene in chrysanthemum was used as the internal expression control.

The transcriptional activity of *CmBES1* was examined using a yeast assay system and a luminescence assay. The ORFs (1 to 918 bp nucleotides) of the *CmBES1* gene were amplified using primers that added *Eco*R I and *Sal* I sites. The primers used are listed in Supplementary Table S4. The PCR products and pGBKT7 vector were digested with *Eco*R I and *Sal* I and then ligated with T4 DNA ligase (TaKaRa, Tokyo, Japan) to produce a pGBKT7-*CmBES1* expression vector. The pGBKT7-*CmBES1*, pCL1 (positive control), and pGBKT7 (negative control) constructs were subsequently transformed into a *Y2H Gold Saccharomyces cerevisiae* strain (Clontech, Tokyo, Japan). The yeast transformants expressing pGBKT7-*CmBES1* or pGBKT7 were incubated on SD/-Trp media at 30 °C for 3 days, and those expressing pCL1 were incubated on SD/-Leu media. The transformed yeast cells were then transferred to SD/-His-Ade media for 8 h in the presence of X-α-gal to screen for positive transformants.

For the luminescence assay, a 35S::GAL4DB-*CmBES1* plasmid was constructed with the pENTR1A-*CmBES1* plasmid (the primers used are listed in Supplementary Table S4) by the LR reaction (Invitrogen). The transient expression assay was performed in Arabidopsis protoplasts as previously described^37^. For each transformation, 5 μg of 35S::GAL4DB-*AtARF5* (positive control), 35S::GAL4DB (negative control) or 35S::GAL4DB-*CmBES1* and 5 μg of 5× GAL4-LUC plasmid (luminescence reporter) were used. The luciferase activity was measured as previously reported^38^. Three independent experiments were performed.

### Genetic transformation of chrysanthemum

The pMDC43-*CmBES1* overexpression plasmid was introduced into WT chrysanthemum Jinba via Agrobacterium-mediated genetic transformation^39^. After screening by PCR with the appropriate vector primers (Supplementary Table S4), positive lines were further analyzed with qRT-PCR using BES1-RT-F/R primers to determine the relative expression levels. Each sample was tested for three biological and three technical replicates. Overexpression and WT chrysanthemum lines during the same growth period were grown in a growth chamber for 1 week under 16 h light/8 h dark day/night conditions at 25 °C/18 °C (day/night) before they were transferred to a greenhouse and subjected to standard management practices.

### Morphological and histological analyses

The outermost ray florets at the open-flower stage of WT and *CmBES1* transgenic chrysanthemum plants were used to measure the CTMD, which was defined as the corolla tube length/ray floret length and measured as described previously^22^. The main characteristics compared between the ray florets and disc florets are listed in Supplementary Table S5. The whole inflorescence (either 2 or 4 mm in diameter) or only the outermost ray florets was sampled and fixed with 2.5% (v/v) glutaraldehyde after removing the bracts. The samples were dried to the critical point and then coated with gold before they were observed by scanning electron microscopy (SU8010 device, Hitachi, Japan). Fresh inflorescence and ray florets were also imaged with an S8AP0 optical microscope (Leica Camera AG, Germany).

### RNA extraction and RNA-seq analysis

The outermost ray florets of WT and *CmBES1* transgenic chrysanthemum were sampled at 4 mm and because of their small size, each sample consisted of nine inflorescences. Total RNA was extracted using an RNA Isolation Kit (Waryong, Beijing, China) and subjected to an Illumina HiSeq™2000 instrument located at the Beijing Genomics Institute (Shenzhen, China; http://www.genomics.cn/index) for sequencing according to the manufacturer’s instructions. Adaptor sequences and low-quality reads were removed from the raw sequence data, and the transcriptomic analysis was conducted with the Trinity program^40^. For unigene annotations, homology searches employed the NCBI nonredundant nucleotide (NT), NCBI nonredundant protein (NR), Swiss-Prot, Kyoto Encyclopedia of Genes and Genomes (KEGG), COG (Cluster of Orthologous Groups), and Pfam databases. In the present analysis, an FDR below 0.05 was identified as the criterion for DEGs^41^.

### Quantitative RT-PCR analysis

To verify the expression characteristics of *CmBES1* and the expression differences of key DEGs in the three transgenic lines and WT chrysanthemum, qRT-PCR analysis was performed. The primers used for qRT-PCR were designed by Primer 5 software (Supplementary Table S4). The reference gene *EF-1α* (GenBank: KF305681) was used as an expression control. Each sample was represented by three biological and three technical replicates. The specific experimental qRT-PCR methods used have been described previously^42^. The relative transcript levels of DEGs were calculated using the 2^−ΔΔCT^ method^43^.

### Statistical analysis

Analysis of variance by Duncan’s multiple range test was used to identify significant differences between genotypes and/or treatments. The statistical analyses were performed by SPSS v17.0 software (SPSS Inc., Chicago, IL).

## Supplementary information

Supplementary Information

Supplementary Information2
