# Supplementary material for: CmBES1 is a regulator of boundary formation in chrysanthemum ray florets
Source: Hortic Res. 2020 Aug 1;7:129. doi: 10.1038/s41438-020-00351-8 (PMC7395151; doi:10.1038/s41438-020-00351-8)
Supplement: Supplementary file 1 — Supplementary Information [file 41438_2020_351_MOESM1_ESM.doc]

**Supplemental material**

**Supplemental Tables**

**Table S1 Summary of sequencing and de novo assembly**

| **Sample** | **WT-1** | **WT-2** | **WT-3** | **OX4-1** | **OX4-2** | **OX4-3** |
| --- | --- | --- | --- | --- | --- | --- |
| **Total Raw Reads (M)** | 67.68 | 65.17 | 67.68 | 67.68 | 67.68 | 65.17 |
| **Total Clean reads (M)** | 65.52 | 63.13 | 65.38 | 65.26 | 65.34 | 62.74 |
| **Total Clean Bases (Gb)** | 6.55 | 6.31 | 6.54 | 6.53 | 6.53 | 6.27 |
| **Clean Reads Q20 (%)** | 97.84 | 97.73 | 97.67 | 97.67 | 97.75 | 97.62 |
| **Clean Reads Q30 (%)** | 90.98 | 90.64 | 90.44 | 90.47 | 90.66 | 90.28 |
| **Clean Reads Ratio (%)** | 96.82 | 96.87 | 96.61 | 96.43 | 96.54 | 96.27 |
| **Total Number of unigenes** | 65,984 | 59,431 | 62,636 | 67,405 | 64,908 | 65,476 |
| **Mean Length of unigenes (bp)** | 773 | 756 | 795 | 825 | 798 | 802 |
| **N50** | 1,091 | 1,057 | 1,140 | 1,203 | 1,139 | 1,153 |
| **All-Unigene** | 98,155 |  |  |  |  |  |
| **Mean Length (bp)** | 987 |  |  |  |  |  |
| **N50 (bp)** | 1,407 |  |  |  |  |  |

**Table S2 The DEGs related to organ boundary growth and flower development**

**Table S3** The DEGs related to brassinosteroid (BR) biosynthetic pathway

| Gene ID | OX4_1 FPKM | OX4_2 FPKM | OX4_3 FPKM | WT1 FPKM | WT2 FPKM | WT3 FPKM | log2  (OX4/WT) | Description |
| --- | --- | --- | --- | --- | --- | --- | --- | --- |
| CL13153.Contig1_All (BR6ox1) | 12.32 | 14.67 | 17.97 | 9.68 | 8.78 | 11.6 | 0.572097 | *Helianthus annuus* cytochrome P450 85A1-like |
| CL5486.Contig2_All (CPD) | 9.09 | 7.87 | 8.43 | 5.65 | 5.46 | 7.27 | 0.46163 | Cytochrome P450 90A1 |
| Unigene11129_All (ROT3) | 8.81 | 8.3 | 9.26 | 9.54 | 7.86 | 8.17 | 0.039365 | cytochrome P450 superfamily protein |

**Table S4 Primer sequences used in the test**

| **Primer name** | **Primer sequence (5′-3′)** |
| --- | --- |
| CmEF1a-F | TGTAACAAGATGGATGCCACAA |
| CmEF1a-R | TCGCCCTCAAACCCAGAAAT |
| BES1-F | CTCTTCATCATCTGCACCAG |
| BES1-R | CTCTTCATCATCTGCACCAGATTTG |
| BES1-1A-SalI-F | ACGCGTCGACATGACTGGAGGCGGTGCAT |
| BES1-1A-EcoRI-R | CGGAATTC TAGTTGGCGGCTTTACCATT |
| BES1-RT-F | TGGGCCAGGTTCTGAGTTTG |
| BES1-RT-R | GCTCCAGGTCGTCTGTTCCA |
| CmBES1-test-F | GACGCACAATCCCACTATCC |
| CmBES1-test-R | TCTTCAACAACCCAACCAGC |
| IAA13-RT-F | CCAGCGAGTTGAGGCAAAAG |
| IAA13-RT-R | AGCCCAAACGATGAAGGAAA |
| CUC2-RT-F | TTCACTGGTCGAGCTTTTGCT |
| CUC2-RT-R | GGTTGCGCGGTTAGTTCGTA |
| CYC4-RT-F | CGAGATCGAAGGGTGAGATTG |
| CYC4-RT-R | TCAGAAAGATTTGAAGAAGAGGTA |
| SOC1-RT-F | TTAGGAACCAGCACTATCGATGAA |
| SOC1-RT-R | TTTTCTCCTTTGCGTGTAGTTGTT |
| fzqFL-3-RT-F | TTGGCCATGGGTGACAAAAT |
| fzqFL-3-RT-R | TGGCCTGGATCAAAATGGAA |
| AIL6-RT-F | CTTCCACATCATACCGGTTCA |
| AIL6-RT-R | ACAAGGTCGTTGGCAAGCA |
| ROT3-RT-F | ATGGGTCTCAACAACGACTGCC |
| ROT3-RT-R | ATCCACAAGACAAGCAAGAGCTG |
| CPD-RT-F | AGGATGCATTCTTTAACTATGAGTT |
| CPD-RT-R | ATTTAGCCTCACCAACCTGTCT |
| RT-BR6ox1-F | GATAATAATGCACCTCTCATGTTCT |
| RT-BR6ox1-R | GCAAAGGCATACTTCCTGGTTA |

**Table S5 The main characteristics comparation between ray florets and disc** florets

| **Character** | **Ray florets** | **Disc florets** |
| --- | --- | --- |
| Symmetry | bilaterally symmetrical | radially symmetrical |
| Location | peripherally | central |
| Fertility | male sterile | hermaphroditic |
| Stamens | arrested | normal |
| Corolla | two rudimentary dorsal petals and elongated ventral ligule formed by three fused petals | five equivalent petals |

**Supplemental Figures**


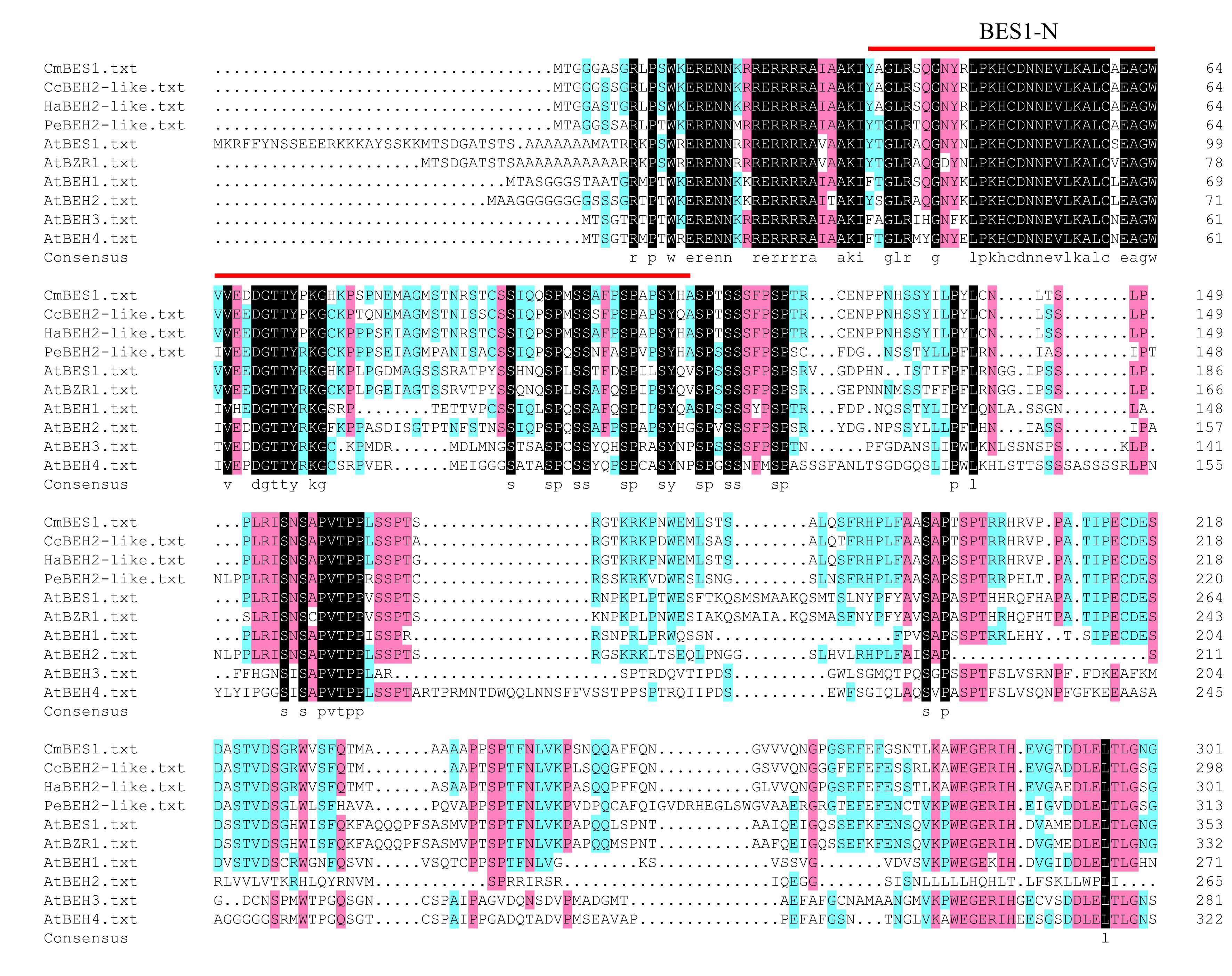


**Fig. S1 Amino acid sequence alignment of CmBES1 with BES1 sequences from other plant species.** Single red lines indicate the conserved BES1-N region in BES1-like proteins. Black represents 100 % identity, red 75 % identity, and blue 50 % identity. The sequence compared to CmBES1 were *Arabidopsis thaliana* *AtBES1* (AT1G19350), *AtBZR1* (AT1G75080), *AtBEH1* (AT3G50750.1), *AtBEH2* (AT4G36780), *AtBEH3* (AT4G18890), *AtBEH4* (AT1G78700), *Helianthus annuus* BES1/BZR1 homolog protein 2-like (*HaBEH2-like*, LOC110908961), *Cynara cardunculus* BES1/BZR1 homolog protein 2-like (*CcBEH2-like*, LOC112512677), and *Populus euphratica* BES1/BZR1 homolog protein 2-like (*PeBEH2-like*, LOC105107647).


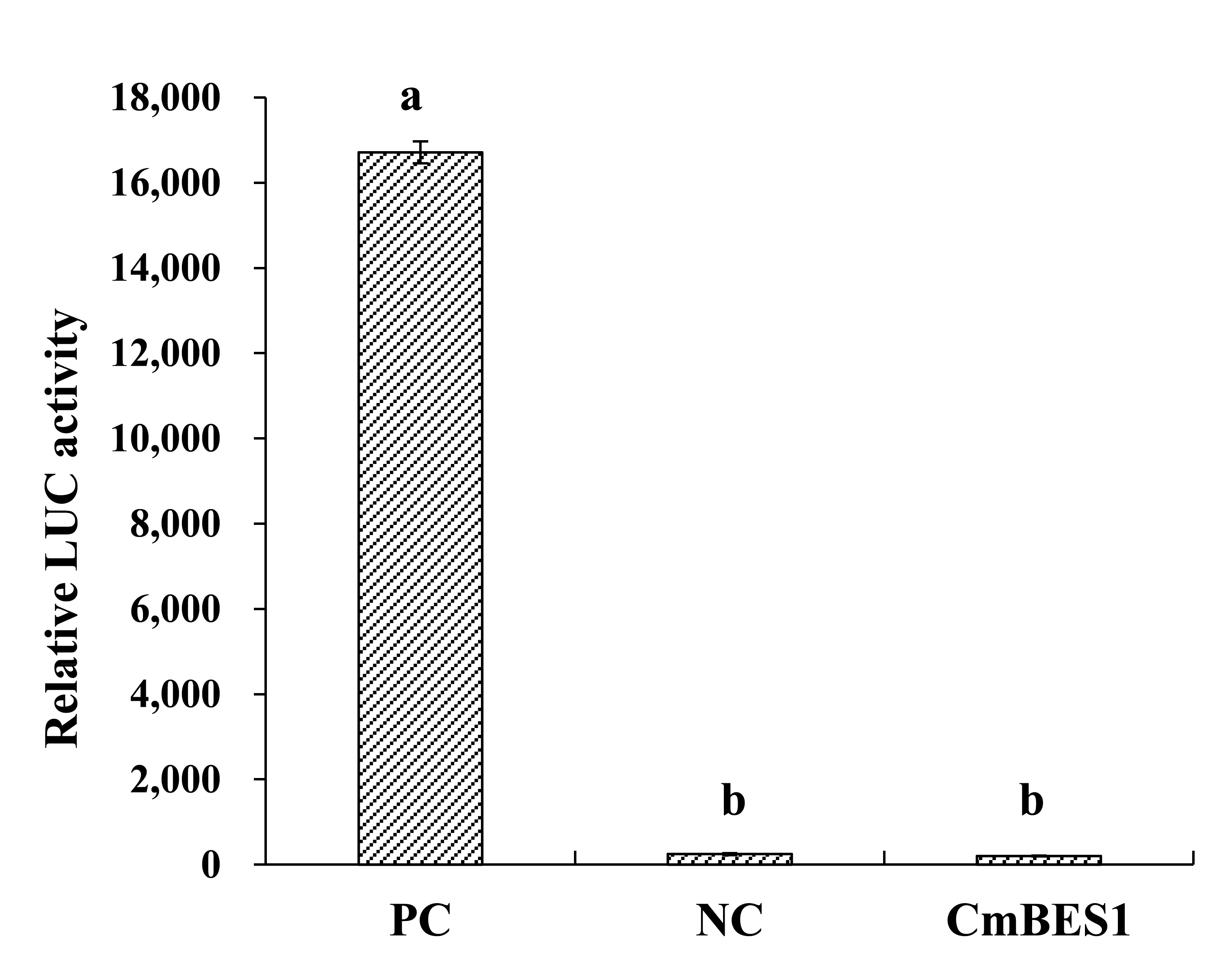


**Fig. S2 Relative luciferase activities in Arabidopsis mesophyll protoplasts transfected with 35S::GAL4DB-CmBES1.** Error bars indicate SE (n = 3). Differences were analyzed by Duncan’s multiple range test. Different lowercase letters indicate signiﬁcantly differences (*P* < 0.01).


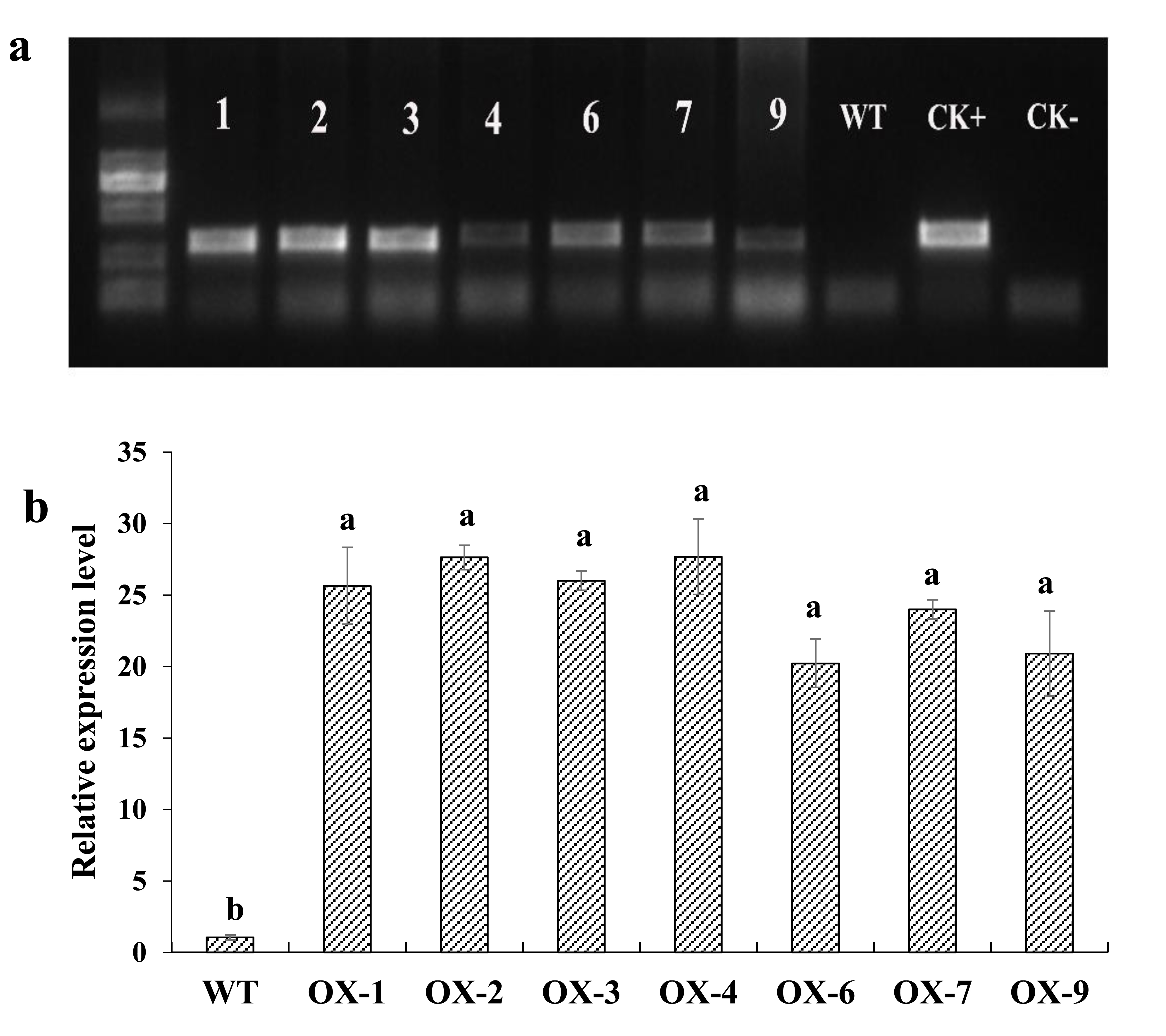


**Fig. S3 Analyses of *CmBES1*-overexpressing transgenic lines. a** PCR analyses of *CmBES1* transgenic lines. M: DL2000; Wild Type: ‘Jinba’; 1-9: Resistance lines; ck+: Positive control; ck-: Negative control. **b** Relative expression levels of *CmBES1* in WT and OX transgenic lines. Error bars indicate SE (n = 3). Differences were analyzed by Duncan’s multiple range test. Different lowercase letters indicate signiﬁcantly differences (*P* < 0.05).


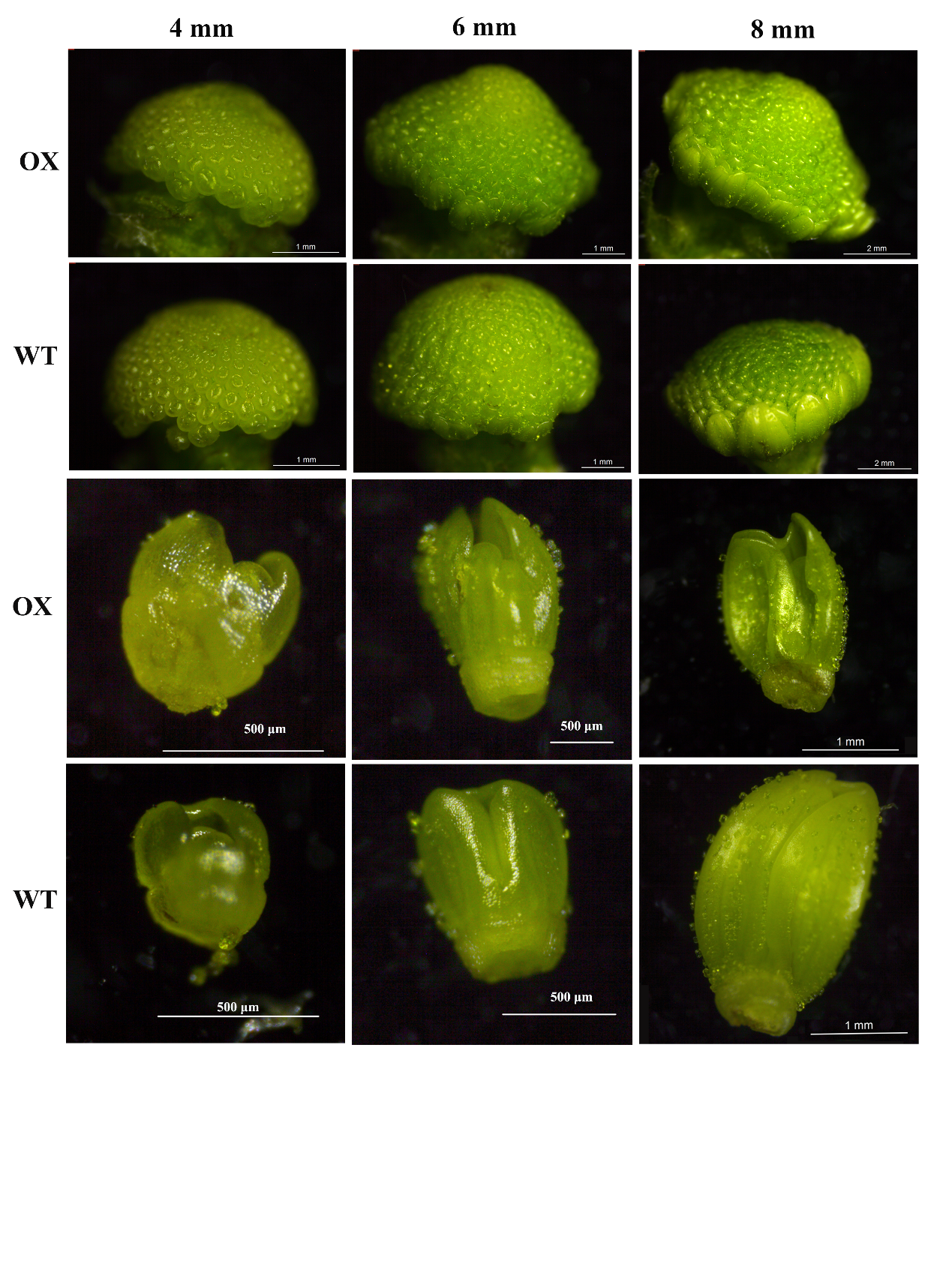


**Fig. S4 Morphological analysis of wild type and *CmBES1* overexpressing transgenic line inflorescences at the 4 mm, 6 mm, and 8 mm stage.**


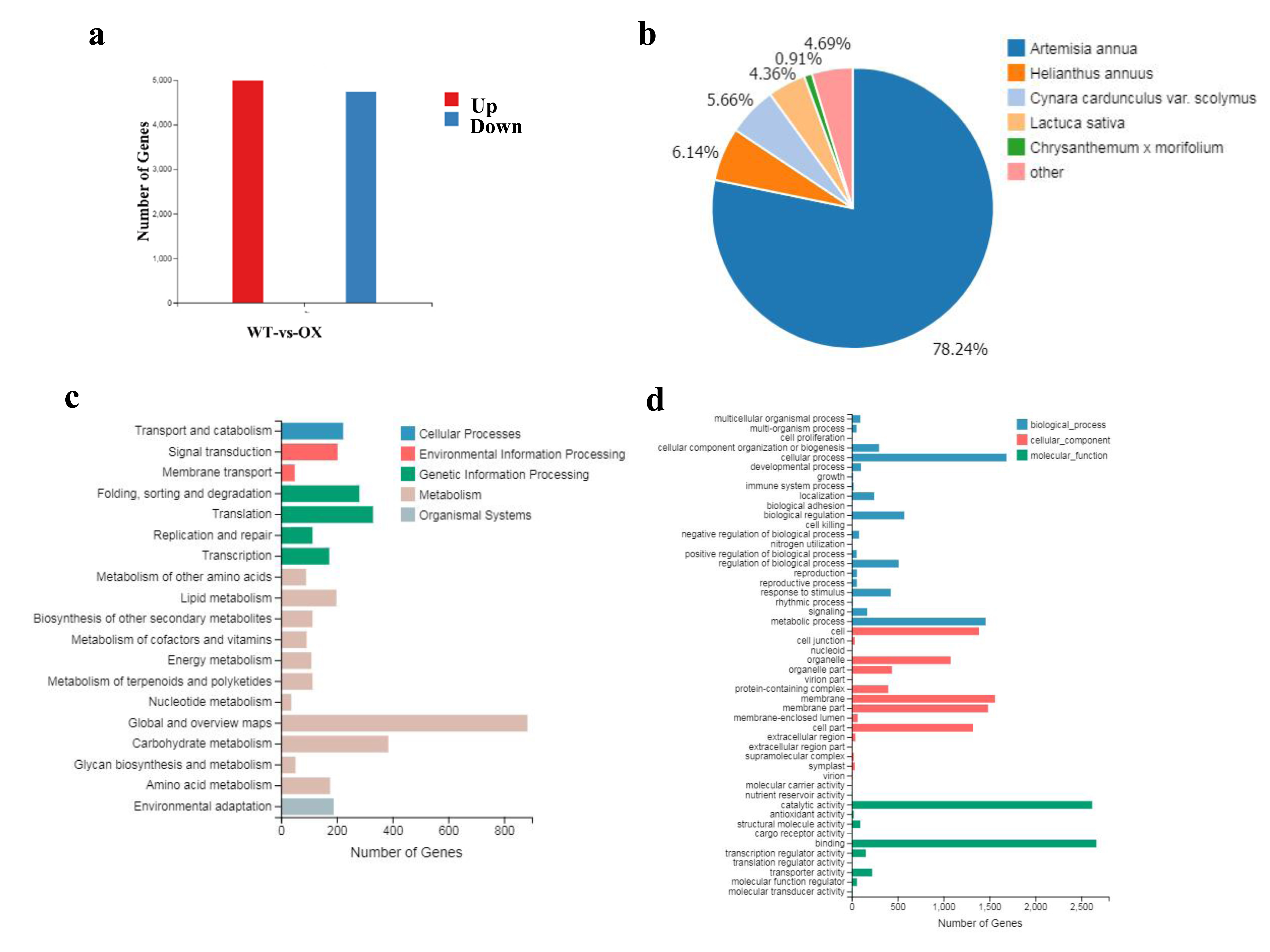


**Fig. S5 Summary of sequencing data**. **a** Differentially expressed genes (DEGs) identified in pairwise comparisons between wild type and *CmBES1* overexpressing transgenic line libraries. **B** Species used for the gene sequence analysis comparison, *Artemisia annua*, *Helianthus annuus*, *Cynara cardunculus var. scolymus*, *Lactuca sativa*, *Chrysanthemum morifolium*. **c** KEGG annotation of the Unigene set. **d** GO classification of the Unigene.


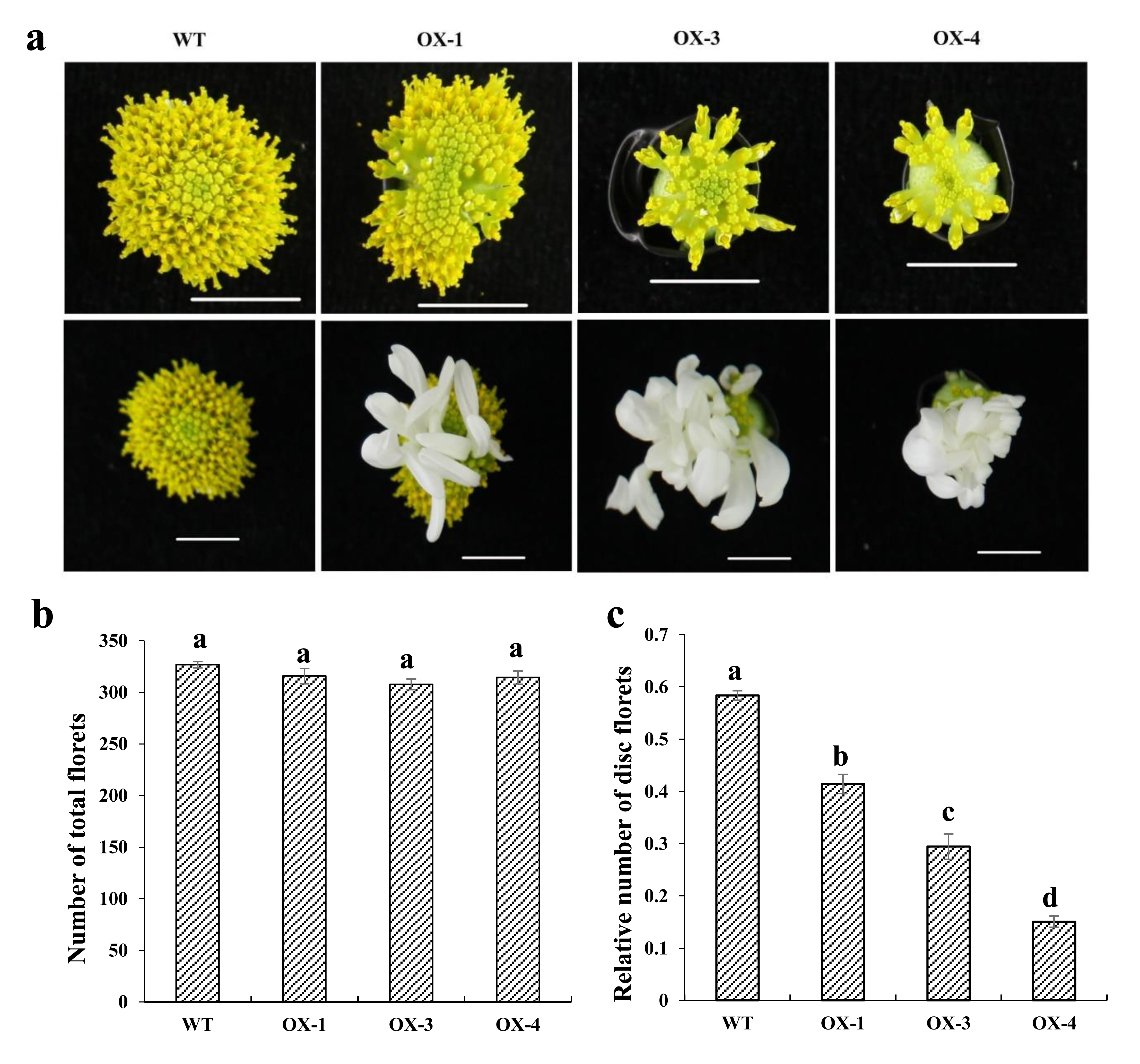


**Fig. S6 Ectopic expression of *CmBES1* affects the number of ray and disc floret. a** Top view of disc florets in wild type (WT) and transgenic overexpressing (OX) lines 1, 3, 4. **b** Total number of florets in the capitulum between WT and OX lines. **c** The relative number of disc florets between WT and OX lines. Error bars indicate SE (n = 12). Differences were analyzed by Duncan’s multiple range test. Different lowercase letters indicate signiﬁcantly differences (*P* < 0.01).


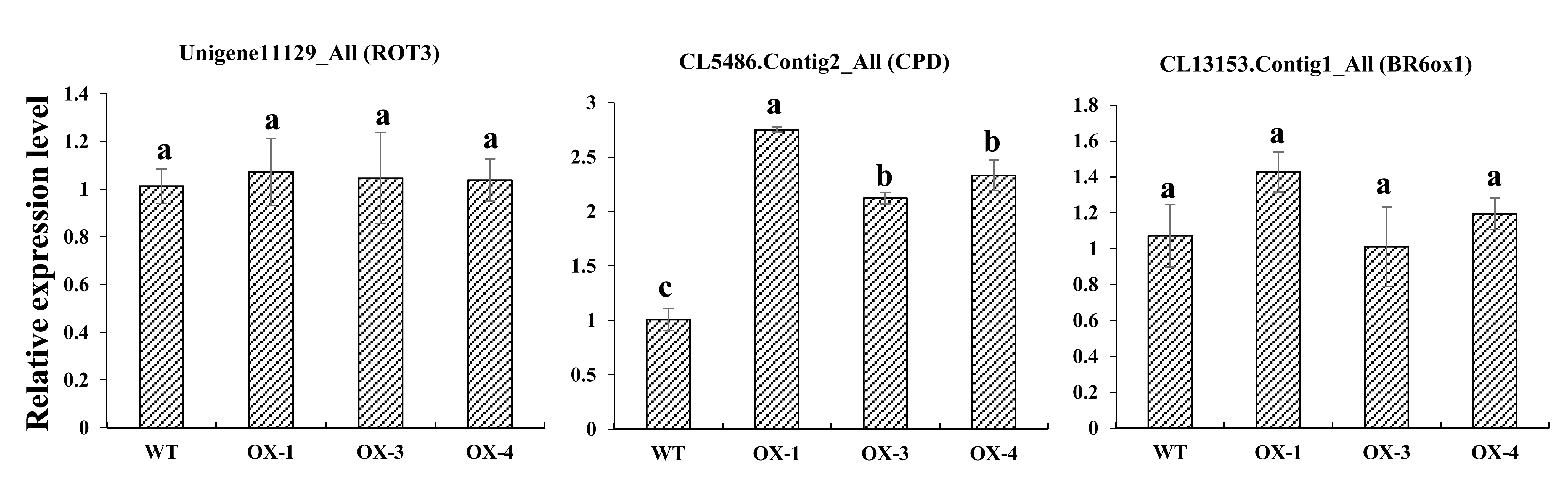


**Fig. S7** **Validation of the RNA-Seq classification of brassinosteroid (BR) biosynthetic pathway genes using qRT-PCR**. Error bars indicate SE (n = 3). Differences were analyzed by Duncan’s multiple range test. Different lowercase letters indicate signiﬁcantly differences (*P* < 0.05).
